# P300 amplitude variation is related to ventral striatum BOLD response during gain and loss anticipation: An EEG and fMRI experiment

**DOI:** 10.1016/j.neuroimage.2014.03.077

**Published:** 2014-08-01

**Authors:** Daniela M. Pfabigan, Eva-Maria Seidel, Ronald Sladky, Andreas Hahn, Katharina Paul, Arvina Grahl, Martin Küblböck, Christoph Kraus, Allan Hummer, Georg S. Kranz, Christian Windischberger, Rupert Lanzenberger, Claus Lamm

**Affiliations:** a Social, Cognitive and Affective Neuroscience Unit, Department of Basic Psychological Research and Research Methods, Faculty of Psychology, University of Vienna, Vienna, Austria; b Center for Medical Physics and Biomedical Engineering, Medical University of Vienna, Vienna, Austria; c Department of Psychiatry and Psychotherapy, Medical University of Vienna, Vienna, Austria

**Keywords:** Anticipation, Reward, Monetary incentive delay task, fMRI, EEG, Motivation

## Abstract

The anticipation of favourable or unfavourable events is a key component in our daily life. However, the temporal dynamics of anticipation processes in relation to brain activation are still not fully understood.

A modified version of the monetary incentive delay task was administered during separate functional magnetic resonance imaging (fMRI) and electroencephalogram (EEG) sessions in the same 25 participants to assess anticipatory processes with a multi-modal neuroimaging set-up.

During fMRI, gain and loss anticipation were both associated with heightened activation in ventral striatum and reward-related areas. EEG revealed most pronounced P300 amplitudes for gain anticipation, whereas CNV amplitudes distinguished neutral from gain and loss anticipation. Importantly, P300, but not CNV amplitudes, were correlated to neural activation in the ventral striatum for both gain and loss anticipation. Larger P300 amplitudes indicated higher ventral striatum blood oxygen level dependent (BOLD) response.

Early stimulus evaluation processes indexed by EEG seem to be positively related to higher activation levels in the ventral striatum, indexed by fMRI, which are usually associated with reward processing. The current results, however, point towards a more general motivational mechanism processing salient stimuli during anticipation.

## Highlights

• Monetary incentive delay task was assessed in separate EEG and fMRI sessions.
• Both gain and loss anticipation activated well known reward-related areas.
• P300 amplitude differentiated reward cue from non-reward cues.
• P300 amplitudes positively correlated with BOLD response in the ventral striatum.
• Ventral striatum activation associated with general motivational processes.

## Introduction

Waiting for a loved-one to return or being afraid of losing his or her affection after a long period of separation — the anticipation of favourable or unfavourable events is a key component of our daily life. Our wellbeing is highly dependent on how we deal with the constant confrontation with positive and negative challenges and their consequences. Thus, understanding the neural basis of the cognitive and affective processes associated with reward and loss anticipation in normally functioning individuals is of particular importance when trying to understand mental conditions in which reward-related processing is disrupted.

Reward processing is mainly characterised by two temporally distinct stages — an appetitive (i.e., preparatory or anticipatory) phase is followed by a consummatory phase (Berridge, 1999). The current study focuses on the appetitive phase where potential rewards and losses are present. The appetitive phase is composed of reward anticipation and related motor-preparation processes. The anticipatory affect model (Knutson and Greer, 2008) suggests that the anticipation of positive stimuli leads to positive arousal which in turn promotes approach behaviour, whereas the anticipation of negative stimuli leads to negative arousal promoting avoidance behaviour. So far, research mainly used functional magnetic resonance imaging (fMRI) to study anticipation-related processes. Only a few studies investigated these processes with electroencephalography (EEG). The combination of both methods, which was applied in the current study, allows for multimodal assessment of anticipation-related processes benefitting from the technical advantages of both methods.

Extensive evidence suggests that brain structures such as the midbrain, the ventral striatum including nucleus accumbens (NAcc), amygdala, and orbital mesial parts of the prefrontal cortex are chiefly involved in reward processing (e.g., Arias-Carrion and Poppel, 2007, Liu et al., 2011, McClure et al., 2004, O'Doherty, 2004, Schultz, 2006, Sescousse et al., 2013). The neurotransmitter dopamine is attributed an important role in reward processing (Schultz, 2006). Note however, that the same brain regions which are associated with reward play also an important role during aversive motivation and learning in animal models (Salamone et al., 1994). Therefore, it is still a matter of debate whether these brain networks reflect only reward processing or whether they reflect, in more general terms, a motivational system.

Electroencephalographic components such as the P300 event-related potential (ERP) and the slow wave contingent negative variation (CNV; Walter et al., 1964) have also been implicated in anticipatory reward and motor preparation processes. Both have been previously termed as putative reward-related electrophysiological markers (Goldstein et al., 2006) and have been described to be evoked during the anticipatory phase of an electrophysiological monetary incentive delay (MID) task in which participants can win or lose money after being cued whether monetary gain or loss is possible in the current trial (Broyd et al., 2012, Santesso et al., 2012). The MID task was also used in the current study.

In general, the P300 is a positive-going ERP deflection peaking between 300 and 600 ms after stimulus presentation (Duncan Johnson and Donchin, 1977, Johnson and Donchin, 1980). P300 amplitude variation is related to categorical stimulus probability (Johnson and Donchin, 1980, Kutas et al., 1977), stimulus quality, attention (Polich and Kok, 1995), task relevance (Coles et al., 1995), task complexity (Isreal et al., 1980), and effort spent on a task (Brocke et al., 1997). Moreover, P300 amplitude variation is related to reinforcer magnitude (Goldstein et al., 2006). Thus, whenever task-relevant stimuli are presented during an experiment a positive ERP deflection in the time window around 300 ms post stimulus can be observed, with maxima at midline electrodes. Prominent theoretical accounts relate P300 amplitude variation to context updating in working memory (Bonala and Jansen, 2012, Donchin and Coles, 1988), e.g., updating whether a potential gain or loss is at stake in the MID task.

The CNV is a negative-going potential shift which is primarily associated with anticipatory attention and preparation of effortful processes (Falkenstein et al., 2003, Gómez et al., 2007). The CNV component is assumed to reflect neural activity within the thalamo–cortico–striatal network (Fan et al., 2007, Macar and Vidal, 2003, Pfeuty et al., 2005).

Although these two ERP components are not specific for reward anticipation, they might be indirectly influenced by similar underlying neuronal processes related to dopamine which drive activation patterns during fMRI investigations. Indeed, an association between P300 amplitude variation and central dopamine system has been reported previously (Pogarell et al., 2011, Takeshita and Ogura, 1994). Clinical and genetic studies provide further evidence for a potential contribution of dopaminergic neurotransmitter systems to P300 amplitude variation (Berman et al., 2006, Blackwood, 2000, Houston et al., 2003, Mulert et al., 2006, Oribe et al., 2013). The CNV component has been associated with central dopaminergic activity in a similar vein (Fan et al., 2007, Linssen et al., 2011). Note, however, that these assumptions are based on indirect evidence since actual dopamine transmission is not accessible by neither fMRI nor EEG, as used here.

The current study aimed to further investigate the question whether activation in so-called reward-related brain areas, in particular the ventral striatum, reflects only reward processing or more general motivational processes. To this end, a modified version of the MID task (Knutson et al., 2000), a prototypical cued response task, was administered during separate fMRI and EEG sessions in the same participants. To investigate this question, we performed fMRI and EEG measurements (1) to use fMRI for assessing neural activations in “classical” reward-related brain areas, and (2) to compare these activations to ERP components such as the P300 and CNV. The rationale of this comparison was to investigate whether associations can be found with either an ERP component reflecting aspects of salience and attention during the anticipation process – in particular the P300 component – or with an ERP component reflecting more cognitive effort aspects of the anticipation process – in particular the CNV component. For the imaging data, we expected enhanced neural activation in reward-related brain areas for reward anticipation compared to non-reward and neutral anticipation (Knutson and Greer, 2008, Knutson et al., 2000, Knutson et al., 2003). For the electrophysiological data, we expected a differentiation for reward compared to non-reward and neutral anticipation for P300 and CNV amplitudes (Broyd et al., 2012, Gruber and Otten, 2010). To answer our research question, we combined results of both methods via calculating correlations between electrophysiological amplitude variation and hemodynamic activation in the ventral striatum (Goldstein et al., 2006, Pogarell et al., 2011, Takeshita and Ogura, 1994). Finding a significant correlation for both gain and loss cues between ERPs and ventral striatum BOLD responses would support the general motivational mechanism hypothesis by reflecting that similar underlying mechanisms are engaged during gain and loss anticipation. In contrast, a significant correlation between ERPs and BOLD response solely for gain cues would support the reward hypothesis. Moreover, correlations with P300 vs. CNV amplitudes would indicate different processes. While P300 correlations would be related to salience and attention, correlations with CNV amplitudes would indicate the engagement of processes related to cognitive effort.

## Material and methods

### Participants

Initially, 29 volunteers took part in our experiment. Four participants dropped out during the study due to technical problems with the scanning. The final sample consisted of 25 individuals (13 women) with a mean age of 23.8 years (SD = 3.60). All participants were right-handed as assessed with the Edinburgh Handedness Inventory (Oldfield, 1971), had normal or corrected-to-normal vision, and were screened with the Structural Clinical Interview for DSM-IV (SCID; APA, 1994) to exclude individuals with psychiatric disorders. Moreover, participants reported no metal implants, no past or present substance abuse, no psychopharmacological medication within the last three months, and no pregnancy (tested with urine human chorionic gonadotropin pregnancy test). All participants gave written informed consent prior to data acquisition. The study was approved by the Ethics Committee of the Medical University of Vienna and the General Hospital of Vienna. Participants were reimbursed for their study participation. They participated in further paradigms which are outside the scope of the current manuscript (Hahn et al., 2013).

### Stimuli and task procedures

#### Monetary incentive delay (MID) task

**Fig. 1:**
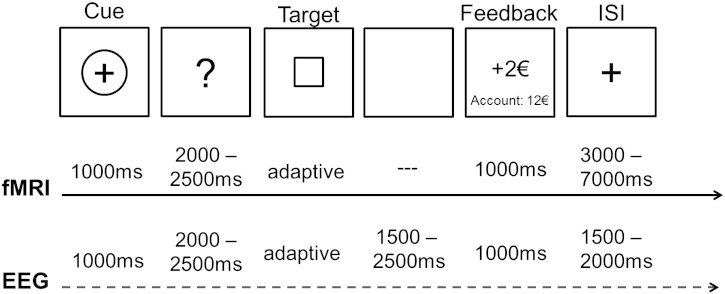
Timeline of the current MID task. fMRI and EEG timing differed slightly since we adapted the MID paradigm for each method appropriately to gain most reliable results. Note that this difference is not relevant for the current study which is solely focusing on the anticipation phase of the MID task.

Participants were administered comparable versions of the MID task (Knutson et al., 2000) for both fMRI and EEG measurements (see Fig. 1). The MID task is designed in a way that participants can maximise rewards and minimise losses by responding as quickly as possible by button press to a visual target. Prior to target presentation, incentive cues are presented to indicate what is at stake in the current trial, i.e., whether responding relates to an attempt to win money, or to avoid losing money, or that no money is at stake. Each trial started with the presentation of the incentive cue for 1000 ms in black colour on a grey background. A potential monetary gain or win was indicated by a circle surrounding a “+” symbol. A potential loss was indicated by a circle surrounding a “−” symbol. Neutral trials in which neither monetary gains nor losses could be incurred were indicated by empty circles. During the subsequent anticipation phase where participants prepared their motor response, the cue symbols were replaced by a question mark presented for a duration that varied in 100 ms steps between 2000 and 2500 ms (uniformly distributed). A black square on a grey background was used as target stimulus. Initially, the target was presented for 264 ms and the participants were required to respond within this time window for a correct response. Participants responded with their right index finger on an MRI compatible response pad for fMRI measurements (Current Designs Inc., Philadelphia, PA, USA) and on button 1 on a standard PC USB keyboard for EEG measurements. Based on individual reaction times for gain, loss, and neutral cue trials, target duration was shortened by 64 ms after correct responses and prolonged by 64 ms after incorrect responses. This adaptive algorithm ensured that participants' accuracy levels were approximately 50% for each incentive cue. Feedback was presented for 1000 ms immediately after the target offset during fMRI measurements and after a variable duration of 1500 to 2500 ms during EEG measurements. Feedback consisted of the monetary gain or loss amount in €, presented centrally on the screen. Below the current trial outcome, the overall amount of accumulated money was presented. A variable inter-trial-interval presenting a fixation cross with a duration of 1500–2000 ms during EEG, and 3000–7000 ms during fMRI (uniformly distributed), was presented before the next trial. Each participant was endowed with 12 € at the beginning of each experiment. In cases where the gain cue was presented and the motor response fell within the target time window, participants won 2 €. In cases where the loss cue was presented and the motor response was too slow, participants lost 2 €. Feedback comprised of 0 € in cases where the neutral incentive cue was presented irrespective of button press, when motor responses were too slow in gain trials, or when motor responses were fast enough in loss trials. During fMRI, 100 trials were presented with 40 gain, 40 loss, and 20 neutral trials, recorded in two runs. During EEG, 200 trials were presented with 80 gain, 80 loss, and 40 neutral trials. Participants completed six training trials prior to each measurement. On average, participants scored 12.92 € (SD = 1.95) in both sessions. The order of the EEG and fMRI sessions was randomly permuted and separated by around 50 days on average.

#### fMRI

Functional MRI measurements took place at the MR Center of Excellence, Medical University of Vienna. Stimulus presentation was controlled by Cogent2000 v.1.29 implemented in Matlab 7.7 (The MathWorks, Inc., Natick, MA). Functional MR images were acquired on a whole-body 3 T Siemens TIM Trio scanner (Siemens Medical Solutions, Erlangen, Germany), equipped with a 32-channel head coil, using a gradient-recalled EPI-sequence with distortion correction (TR = 1.8 s, TE = 38 ms, FA = 60°, voxel size 1.5 × 1.5 × 3 mm, 23 slices, GRAPPA2).

Data were pre-processed using SPM8 (FIL Group, UC London, UK). Default parameters were used for slice-time correction (to the middle slice), motion correction (referenced to the mean image), spatial normalisation to MNI (Montreal Neurological Institute) stereotactic space using an in-house scanner-specific EPI template, and spatial smoothing (8 mm Gaussian kernel). For the single-subject data analysis (“first-level analysis”), one regressor was modelled for each anticipation cue (gain cue, loss cue, neutral cue; duration: jittered between 2500 and 3000 ms), one for target onset (duration: individual response times per trial, see Table 1 for means), and one for each of the five potential outcomes (gain, loss, omitted gain, averted loss, neutral; duration: 1500 ms) and convolved with the default canonical hemodynamic response function implemented in SPM8. Moreover, nuisance regressors of white matter, cerebrospinal fluid and realignment parameters were added to the model (Hahn et al., 2012). The current report focuses on the anticipation cues. Thus, group statistics (“second-level analysis”) were calculated with random effects models in SPM8; t-contrasts were calculated for *gain* > *neutral*, *loss* > *neutral*, and *gain* > *loss* and the reverse contrasts. Results are presented at a voxel-level family-wise error (FWE) corrected threshold of p < 0.05 (minimal cluster size k = 20 voxels).

**Table 1:** Mean reaction times in ms and standard deviation (SD) for the three incentive cues during MR and EEG sessions.

	Gain cue		Loss cue		Neutral cue	
	Mean	SD	Mean	SD	Mean	SD
MR session	218.91	33.93	219.53	26.38	267.91	33.47
EEG session	209.72	25.93	209.03	27.94	235.08	37.17

#### EEG

EEG measurements took place at the Social, Cognitive and Affective Neuroscience Unit, University of Vienna. Stimulus presentation and synchronisation with EEG recording were controlled by E-Prime 2.0 software (Psychology Software Tools, Inc., Sharpsburg, PA). For EEG data collection, participants were seated comfortably in a sound-attenuated chamber about 70 cm in front of a 21″ cathode ray tube monitor (Sony GDM-F520; 75 Hz refresh rate). EEG was recorded from 61 Ag/AgCl electrodes which were embedded equidistantly in an EEG cap (model M10, EASYCAP, GmbH, Herrsching, Germany). Two additional electrodes were placed 1 cm above and below the left eye to record vertical eye movements. A skin-scratching procedure was applied prior to data collection (Picton and Hillyard, 1972) to keep electrode impedances below 2 kΩ, which was individually assessed by an impedance meter. EEG signals were collected with a DC amplifier (NeuroPrax, neuroConn GmbH, Ilmenau, Germany), recording EEG within a frequency range of DC to 250 Hz and sampled at 500 Hz for digital storage.

EEG data were analysed using EEGLAB 6.03b (Delorme and Makeig, 2004) implemented in Matlab 7.10.0. Offline, EEG data were low-pass filtered with a cut-off frequency of 30 Hz (roll-off 6 dB/octave). Subsequently¸ EEG data were re-referenced to linked mastoids and extended infomax independent component analysis (ICA; Bell and Sejnowski, 1995, Lee et al., 1999) was applied to the data to detect eye movement-related artefacts. After discarding independent components attributed to such artefacts, data segments of the three incentive cues were extracted starting 500 ms prior to stimulus onset and extending 3000 ms beyond them (i.e., covering the minimal time period from cue onset to target onset). The mean amplitude in the first 500 ms served as baseline for each trial. The *lindetrend* Matlab function was applied to these data segments to control for slow DC drifts. Subsequently, a semi-automatic procedure for artefact correction was applied to eliminate trials with voltage values exceeding +/− 75 μV in any channel or voltage drifts of more than 50 μV during the whole epoch. Trials marked by the EEGLAB algorithms were rejected only in cases where visual inspection also indicated artefacts. Afterwards, artefact-free epochs were averaged separately for each participant for the three incentive cues (gain, loss, neutral). On average, 68.8 +/− 6.61 trials were averaged for gain cues, 67.8 +/− 5.90 trials for loss cues, and 31.3 +/− 4.49 trials for neutral cues (from the 80, 80, and 40 trials, respectively). Subsequently, mean amplitudes were calculated for P300 amplitudes (time interval 350–600 ms post cue) at electrode site Pz and CNV amplitudes (time interval 650–1000 ms) at electrode sites Fz, Cz, and Pz. The chosen time intervals were based on visual inspection and literature recommendations (Broyd et al., 2012). Parietal P300 mean amplitudes were subjected to a one-way analysis of variance (ANOVA) with the within-subject factor *incentive cue* (gain, loss, neutral). CNV mean amplitudes were analysed using two-way repeated-measures ANOVAs with the additional within-subject factor *electrode site* (Fz, Cz, Pz). Moreover, Pearson correlations assessed the relation between P300 and CNV amplitudes.

#### Behavioural data analysis

Response times were defined as the interval from target onset to button press during target presentation. Trials with response times faster than 50 ms were discarded from further analysis, as they probably indicated too fast reactions not in accordance with the task instruction. Subsequently, mean response times were logarithmised by a natural logarithm function to approach a more Gaussian distribution, and subjected to a two-way repeated-measures ANOVA with the within-subject factors *measurement session* (fMRI, EEG) and *incentive cue* (gain, loss, neutral).

#### Combined analysis of fMRI and EEG data

The relationship between fMRI and EEG components was assessed by calculating Pearson correlations between activations in regions of interest (ROIs) and the ERP of interest. Anatomical and functional ROIs were created. For the anatomical ROIs, a bilateral anatomical mask for the ventral striatum was defined by a conjunction of the “caudate head” template provided by the WFU-Pick Atlas (Version 3.3, Wake Forest University, School of Medicine, Winston-Salem, North Carolina; www.ansir.wfubmc.edu) and the “accumbens” template taken from the Harvard–Oxford Subcortical Structural Atlas, implemented in FSL (Plichta et al., 2012). For the functional ROIs, MarsBaR v0.43 SPM toolbox was used to create spheres of 10 mm diameter centred at significant peak voxel activation foci in the striatum for the two contrasts *gain* > *neutral* and *loss* > *neutral*. More precisely, for the *gain* > *neutral* contrast, two functional ROIs were created for activation sub-maxima in the left (x/y/z = − 10/8/–6 mm, MNI-space) and right (10/2/–2 mm) ventral striatum. For the *loss* > *neutral* contrast, also two functional ROIs were created in the left (− 10/8/–6 mm) and right (10/2/0 mm) ventral striatum. Since both ROIs in the left as well as both ROIs in the right ventral striatum were mostly overlapping, they were combined with MarsBaR into one ventral striatum ROI per hemisphere. The bilateral anatomical ROIs reflect hypothesis-driven activation in the reward system (Knutson et al., 2001), whereas the functional ROIs reflect data-driven activation patterns specific for the current sample. Thus, the anatomical and functional ROIs were created to validate the current results in both ways.

Mean activation levels of the top 20% of all activated voxels were extracted using the ExtractVals Toolbox version 1.0 implemented in Matlab of the contrasts *gain* > *neutral* and *loss* > *neutral* for both anatomical and functional ROIs (Mitsis et al., 2008). Subsequently, Pearson correlations were calculated between mean activation levels of all ROIs for the *gain* > *neutral* contrast und the gain cue ERPs and between the mean activation levels of structural and functional ROIS for the *loss* > *neutral* contrast and the loss cue ERPs. We used absolute ERP values instead of difference waves, which would be an analogue to the fMRI contrasts, for the correlations between ERPs and BOLD signal because the neutral cue condition was not considered a control condition during our EEG measurement. We also calculated these Pearson correlations with fMRI baseline contrasts to validate the correlations between absolute ERPs and the differential contrasts (see Supplemental material). CNV amplitudes were assessed at Fz, while P300 amplitudes at Pz.

The significance level was set at p < 0.05 for all statistical tests. If necessary, degrees of freedom were adapted with the Greenhouse–Geisser correction. Significant ANOVA main effects were explored with PASW multiple comparisons, and significant interaction effects with Statistica Tukey HSD post-hoc tests. Partial eta-squared (η_p_^2^) is reported to indicate effect sizes for significant ANOVA results. Values of η_p_^2^ = 0.01, η_p_^2^ = 0.06, and η_p_^2^ = 0.14 represent small, medium, and large effects (Kirk, 1996). Statistical analyses were performed using PASW 18 (SPSS Inc., IBM Corporation, NY) and Statistica 6.0 (StatSoft Inc., Tulsa, OK).

## Results

### Behavioural results

Two participants failed to respond to the neutral cues during fMRI measurements. The response time ANOVA revealed main effects of *measurement session* (F(1,22) = 12.36, p = 0.002, η_p_^2^ = 0.36) and *incentive cue* (F(2,44) = 105.30, p < 0.001, η_p_^2^ = 0.83) and an interaction of both factors (F(2,44) = 12.32, p < 0.001, η_p_^2^ = 0.36). Tukey post-hoc tests showed that response times were generally slower after the neutral cue than the two valenced cues (all p-values < 0.008). Moreover, participants responded slower to the neutral cue in the fMRI than in the EEG session (p = 0.001). No differences in response times between fMRI and EEG measurements were observed for any of the valenced cues (all p-values > 0.07). Mean response times are depicted in Table 1.

### fMRI results

**Fig. 2:**
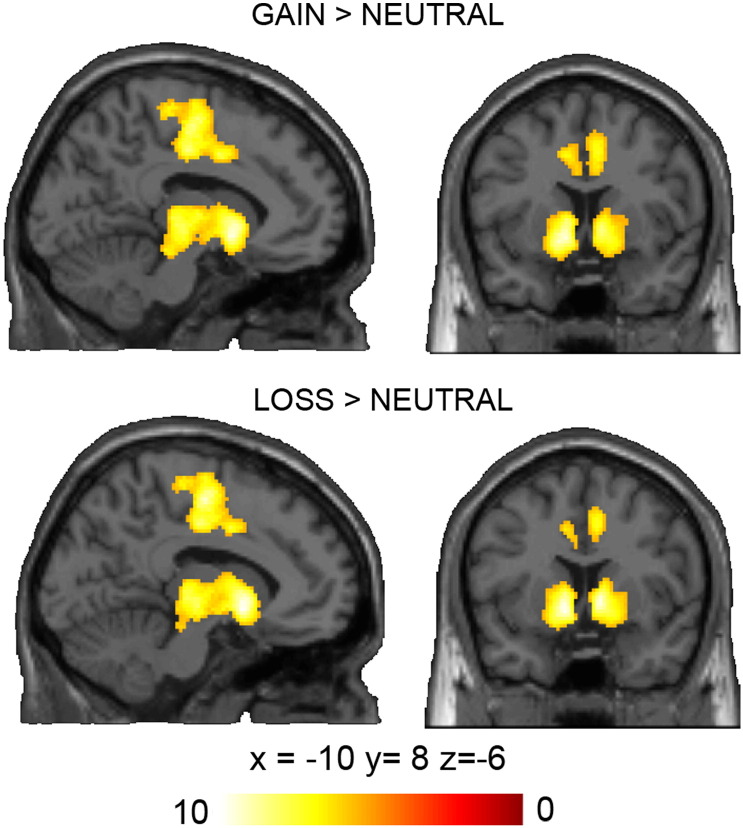
Visualisation of fMRI results showing enhanced neural activation patterns during gain and loss anticipation in areas related to reward processing and motor preparation. Results are presented at a voxel-level family-wise error (FWE) corrected threshold of p < 0.05 (minimal cluster size k = 20 voxels).

Significant activation patterns are displayed in Table 2 and Fig. 2.

**Table 2:** Significant brain activation clusters during anticipation for the contrasts *gain* > *neutral* and *loss* > *neutral* are given including cluster size (k), t-values, and MNI coordinates.

Contrast	Region	k	t max	Coordinates		
				X	Y	Z
Gain > neutral	Brainstem L	4129	10.49	− 6	− 26	− 10
	Ventral striatum L		9.82	− 10	8	− 6
	Ventral striatum R		9.25	10	2	− 2
	SMA R	6283	9.91	32	− 10	46
	Precentral gyrus L		9.51	− 6	− 12	56
	SMR L		9.28	− 36	− 10	50
	Fusiform gyrus R	347	8.36	28	− 86	− 8
	Cerebellum	510	7.46	0	− 56	− 14
			7.27	10	− 60	− 16
			6.79	24	− 52	− 24
	Occipital gyrus L	203	7.25	− 30	− 88	− 8
	Insula R	65	5.92	32	26	0
Loss > neutral	Ventral striatum R	4279	10.35	10	2	0
	Ventral striatum L		10.03	− 10	8	− 6
	Brainstem L		9.69	− 6	− 20	0
	Midcingulate cortex L	4301	7.24	− 8	− 12	56
	SMA L		6.86	− 34	− 10	50
	Midcingulate cortex L		6.67	− 6	− 10	48
	Precentral gyrus R	601	9.62	32	− 10	46
			7.02	54	− 4	48
			6.84	46	− 6	44
	Cerebellum	106	7.02	6	− 56	− 24
			6.55	10	− 60	− 16
	Occipital gyrus R	137	6.87	28	− 86	− 8
			6.06	18	− 90	− 8
	Occipital gyrus L	142	6.69	− 28	− 90	− 8
			6.58	− 20	− 92	− 8
	Lingual gyrus R	32	6.3	18	− 56	− 8
	Superior frontal gyrus R	25	5.72	22	− 6	64

Contrasting *gain* > *neutral* during the presentation of the incentive cue and the subsequent anticipation phase was associated with activation in reward and motor preparation regions such as bilateral ventral striatum and thalamus, bilateral primary motor as well as premotor and supplementary motor cortices. Further large clusters comprised primary and secondary visual areas in both hemispheres as well as cerebellar activation. Additionally, the right insula was activated. Contrasting *loss* > *neutral* yielded mostly comparable results. Large activation clusters were observed bilaterally in the ventral striatum and the thalamus, bilaterally in motor and premotor areas, as well as in the left midcingulate gyrus and the right anterior cingulate gyrus. Furthermore, clusters in the primary and secondary visual areas and the cerebellum were found. Both reverse contracts (*neutral* > *gain*; *neutral* > *loss*) did not reveal any significant results. When contrasting *gain* > *loss*, no significant results were observed either, and the same was the case for the reverse contrast *loss* > *gain*.

### EEG results

**Fig. 3:**
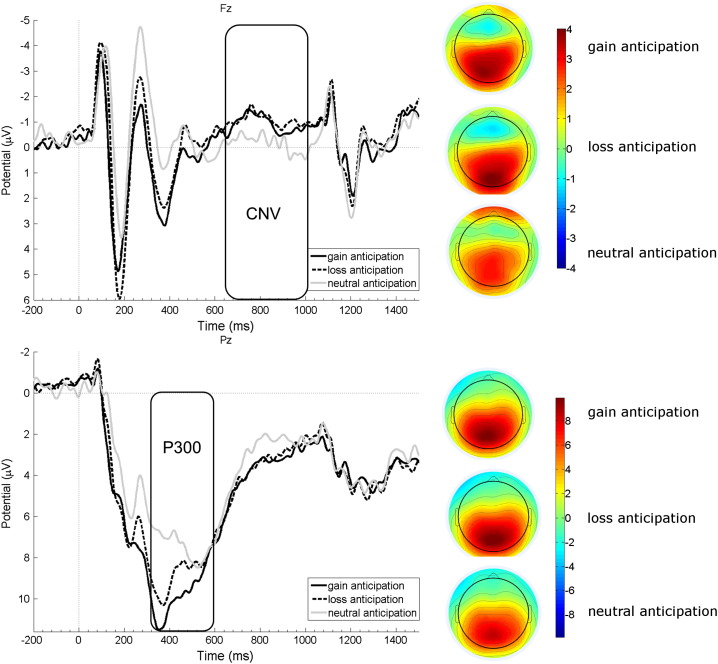
Left side: EEG results displaying CNV amplitude courses of the three incentive cues at Fz in the upper panel and P300 amplitude modulation at Pz in the lower panel. Incentive cue onset is at 0 ms; negative is plotted upwards per convention. Rectangles denote the respective time windows for analyses. Right side: scalp topographies of the CNV component in the upper panel depicting mean activation in the time window 650–1000 ms after stimulus onset. The lower panel depicts scalp topographies of mean activation in the time window 350–600 ms after stimulus onset for the P300 component.

Amplitude time-courses for P300 and CNV components are depicted in Fig. 3.

The one-way ANOVA of P300 amplitudes at Pz revealed a main effect for *incentive cue* (F(2,48) = 7.89, p = 0.003, η_p_^2^ = 0.247). Multiple comparisons indicated that gain incentive cues yielded the most positive P300 amplitudes compared to loss (p = 0.016) and neutral (p = 0.002) cues. In contrast, loss and neutral incentive cues did not differ from each other (p = 0.066), although P300 amplitudes were by trend more positive after loss than neutral cues.

The two-way ANOVA of CNV amplitudes revealed a significant main effect of *electrode* (F(2,48) = 67.59, p < 0.001, η_p_^2^ = 0.74), and no main effect of *incentive cue* (F(2,48) = 0.60, p = 0.941), but a significant interaction (F(4,96) = 7.40, p = 0.001, η_p_^2^ = 0.236). Tukey post-hoc tests indicated that gain and loss cues did not differ at the three electrode locations (all p-values > 0.998). However, the neutral cue elicited more positive amplitudes than the loss cue at Fz (p = 0.009), and more negative amplitudes than the gain cue at Pz (p = 0.025). In general, CNV amplitudes were most negative at Fz compared to Cz and Pz (all p-values < 0.001).

No significant correlations were obtained between P300 and CNV amplitudes for the gain condition (all p-values > 0.113). For the loss and the neutral condition, significant correlations were observed between P300 and CNV amplitudes, at electrode Pz (r = 0.504, p = 0.010 and r = 0.592, p = 0.002, respectively).

**Fig. S1:**
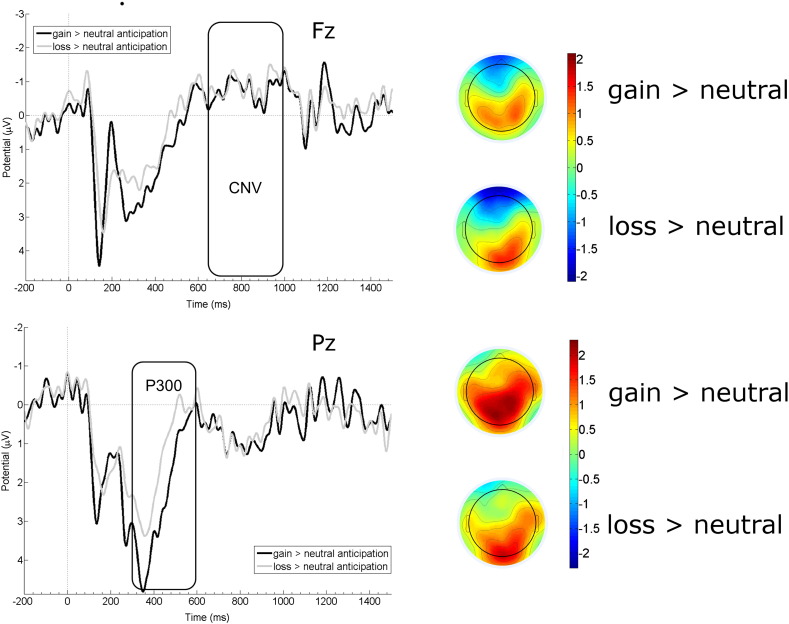
Left side: Difference wave amplitude courses for the comparisons gain cue > neutral cue and loss cue > neutral cue are depicted for CNV amplitudes at Fz (upper panel) and for P300 amplitudes at Pz (lower panel). Rectangles denote the respective time windows for analyses. Right side: Scalp topographies of the difference between gain cue > neutral cue and loss cue > neutral cue for the mean activation in the time window 650–1000 ms after stimulus onset for CNV component (upper panel), and in the time window 350–600 ms after stimulus onset for P300 component (lower panel).

Inline Supplementary Figure S1

### ROI and correlation analyses

Correlation analysis with the bilateral anatomical ventral striatum template revealed significant Pearson correlations between mean P300 gain cue amplitudes and mean activation levels for the contrast *gain* > *neutral* in the left (r = 0.425, p = 0.034) and right (r = 0.415, p = 0.039) ventral striatum ROIs. Mean P300 loss cue amplitudes correlated significantly with the mean activation levels for the contrast *loss* > *neutral* in the left (r = 0.438, p = 0.029) and right (r = 0.414, p = 0.040) ventral striatum ROIs. Correlations between mean P300 neutral cue amplitudes and mean activation levels for anatomical ROIs did not reach significance (all p-values > 0.086). Fisher's test for independent correlations showed that all P300/BOLD correlations here did not differ from each other (all p-values > 0.624).

**Fig. 4:**
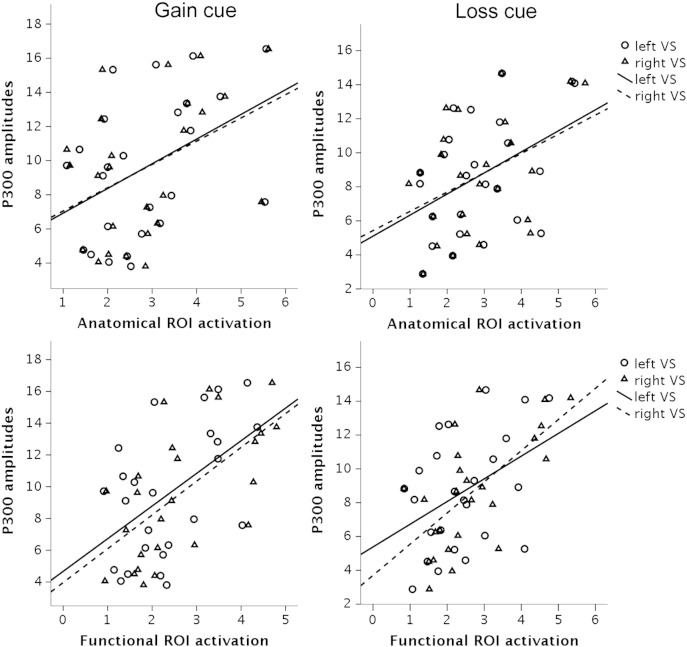
Scatter plots including regression lines of P300 mean amplitudes for gain (left panel) and loss (right panel) incentive cues and mean activation levels for anatomical (upper row) and functional (lower row) ROIs in the ventral striatum. Circles denote left ventral striatum activation, and triangles right ventral striatum activation.

Correlation analysis with the functional ROIs showed similar but stronger associations than anatomical ROIs. Significant Pearson correlations were observed between mean P300 gain cue amplitudes and mean activation levels for *gain* > *neutral* in the left (r = 0.516, p = 0.008) and right (r = 0.621, p = 0.001) ventral striatum ROIs. Moreover, mean P300 loss cue amplitudes correlated significantly with mean activation levels for *loss* > *neutral* in the left (r = 0.420, p = 0.037) and right (r = 0.767, p < 0.001) ventral striatum ROIs, see Fig. 4. However, for functional ROIs significant correlations emerged between P300 neutral cue amplitudes and mean activation levels for the right VS for both *gain* > *neutral* (r = 0.448, p = 0.025) and *loss* > *neutral* (r = 0.405, p = 0.045) contrasts.

No significant correlations emerged for CNV gain, loss, or neutral cue amplitudes and mean activation levels in neither anatomical nor functional ROIs for *gain* > *neutral* (all p-values > 0.122) and *loss* > *neutral* (all p-values > 0.134).

## Discussion

The current study aimed to investigate whether activation in reward-related brain areas during anticipation processes reflects solely reward processing, or could be attributed to more general motivational processes.

Our imaging data revealed a large set of activated areas typically implicated in reward processing according to the literature (Arias-Carrion and Poppel, 2007, McClure et al., 2004, Schultz, 2006). However, contrary to the meta-analysis by Knutson and Greer (2008), but in line with more recent accounts (Carter et al., 2009, Diekhof et al., 2012, Enzi et al., 2012, Liu et al., 2011), the anticipation of the loss incentive cue also yielded strong activation in these so-called “reward-related” areas. The current results speak against the MID task interpretation according to the anticipatory affect model (Knutson and Greer, 2008), which proposed insula activation during loss anticipation. More specifically, our results indicate that the loss incentive cue induced motivation to avoid a potential loss, an interpretation in line with more recent evidence (Broyd et al., 2012, Carter et al., 2009, Diekhof et al., 2012). The association between reward and avoidance of loss or punishment has been intensively investigated in animal studies. Interestingly, some avoidance reactions during punishment anticipation are similar to approach responses during reward anticipation (Ikemoto and Panksepp, 1999). However, others can be accounted for by two-factor theories of avoidance behaviour (Maia, 2010, Mowrer, 1947) proposing that classical as well as instrumental conditioning processes are involved in the development of avoidance behaviour. The notion of comparable processes for gain and loss incentive cues is further supported by comparable reaction times for both incentive cues during fMRI and EEG sessions. Thus, we assume that both incentive cues have predominantly evoked motivation to successfully perform the subsequent motor response.

For the EEG data, we observed significantly enhanced P300 amplitudes after gain incentive cues compared to loss and neutral incentive cues and by trend after loss compared to neutral cues. This finding is in line with previous accounts relating P300 amplitude in a linear way to reinforcer magnitude (Goldstein et al., 2006) or to positively-valenced salient feedback events (Bellebaum et al., 2010a, Gruber and Otten, 2010, Pfabigan et al., 2011). This differentiation between the gain incentive cue and the two others was not observable for CNV amplitudes. In line with the fMRI results, CNV amplitudes only differed between the two valenced cues and the neutral one indicating comparable orienting responses for the two incentive cues. Thus, apart from the initial evaluation differentiating reward from non-reward reflected in P300 amplitudes, subsequent anticipatory ERPs for gain and loss incentive cues were remarkably similar during EEG measurement. This might reflect common sensory and motor requirements of the upcoming motor response for these cues (Löw et al., 2008), pointing towards comparable motivational processes to achieve a reward or to avoid a loss. Apart from reflecting reinforcer magnitude and stimulus salience, P300 amplitude variation has been mostly associated with context-updating in working memory (Bonala and Jansen, 2012, Donchin and Coles, 1988). This model has been expanded by Nieuwenhuis et al. (2005) who proposed that P300 amplitude variation reflects activation of the neocortical locus coeruleus norepinephrine system. At first sight, this proposal seems contradictory to our claim of P300 amplitude relation to dopamine activation. However, concerning neuronal generators, multiple P300 generators have been identified in different cortical and sub-cortical regions (Brázdil et al., 2005) which would suggest that P300 amplitude variation is modulated by several neurotransmitter systems. In addition, different task requirements might activate one neurotransmitter system more than the other. One might speculate that attention-related P300 paradigms rely more on norepinephrine than on dopamine transmission, whereas anticipation- and reward/salience-related P300 paradigms rely more on dopamine than on norepinephrine transmission. Since the current study focused on the anticipation phase of the MID task and because of the observed correlations between P300 amplitudes and ventral striatum activation, we think that our data would speak for dopamine-related P300 modulation.

Interestingly, Fig. 3 shows a pronounced negative deflection prior to the P300 peaks at both electrodes. One might speculate that this ERP is a Feedback-Related Negativity component (FRN; Miltner et al., 1997) elicited by the predictive cue indicating unfavourable events. We performed additional analyses on this negative component applying the same ANOVA model as before (see Supplemental material for a detailed description). Surprisingly, the neutral cue elicited the most negative amplitude deflections compared to negative and positive cues. Previous literature would rather suggest that the negative cue should elicit the most pronounced negativity (Hajcak et al., 2005, Hajcak et al., 2006, Miltner et al., 1997). Therefore, it is not conceptually clear whether the current negative deflection in the respective time range is an FRN component or rather a frontal N2 component reflecting visual template matching or aspects of cognitive control (Folstein and Van Petten, 2008). Therefore, we did not include this ERP in the correlational analysis although FRN amplitudes are assumed to be modified by phasic changes of dopamine in the mesencephalon (Holroyd and Coles, 2002).

Thus, both BOLD response and CNV amplitude variation suggest comparable valence-independent motivational processes. Although there are several studies implying anatomical and functional connections between brain areas related to P300 and CNV components and reward processing, the underlying mechanism driving this association is still a matter of debate. Several studies indicate that the P300 component is associated with motivational processes indexed by reward processing. Mostly, P300 amplitude variation in the context of reward processing was observed after feedback presentation in relation to stimulus valence (Bellebaum et al., 2010b, Pfabigan et al., 2011) and reward magnitude (Yeung and Sanfey, 2004). Additionally, Goldstein et al. (2006) investigated the impact of sustained anticipation of different monetary rewards on P300 and CNV amplitudes. The authors observed significantly larger P300 amplitudes for cues indicating larger gains compared to cues indicating smaller gains at posterior electrodes, but no effect on CNV amplitudes. Multiple neuronal generators in cortical and subcortical areas have been found to account for P300 amplitude variation (Ardekani et al., 2002, Brázdil et al., 2005, Halgren et al., 1998, McCarthy and Wood, 1987). Additionally, regions in the basal ganglia have been reported to contribute to P300 amplitude variation (Rektor et al., 2004). However, note that it is mostly the frontal P3a component which is assumed to be generated in the basal ganglia (for reviews see Linden, 2005, Polich, 2007, Polich and Criado, 2006), and not the posterior P3b component which is more similar to the P300 component investigated in the current study. The differential correlational results suggest that rather processes related to salience (P300 component) but less cognitive effort processes (CNV component) were activated during the anticipation phase. The observed correlations in the current experiment suggest that P300 amplitude variation and ventral striatum BOLD response represent similar mechanisms during incentivised anticipation processes. Since BOLD responses were comparable for gain and loss anticipation and show robust correlations with P300 amplitudes, we assume that they reflect general motivational processes during the anticipation phase and not solely reward-related processes. Beyond that, P300 amplitude variation was also valence-dependent. However, this P300 valence modulation was not comparably reflected in striatal BOLD response (i.e., P300-BOLD correlations did not differ for gain and loss cue anticipation). One might therefore speculate that the observed EEG–fMRI correlations only explain the specific portions of P300 amplitude variation related to motivation, while the additional information on valence contained in P300 must originate from other neural structures (or from processes not captured by BOLD responses). Moreover, since CNV amplitude variation was not related to ventral striatum BOLD responses, we assume that CNV reflects other processes (such as cognitive effort) than motivation during anticipation.

Our data suggest that reward processing is only one aspect of motivational functions of mesolimbic dopamine systems. Accordingly, Salamone and Correa (2012) emphasise that it is an overgeneralisation to relate dopamine neurons exclusively to reward processing. The authors suggest that appetitive and aversive motivational processes such as behavioural activation, exertion of effort, approach behaviour, sustained task engagement, Pavlovian processes, and instrumental learning are all, to some extent, dopamine-related. Thereby, Salamone and Correa (2012) also favour the view that the so-called reward-related brain circuits reflect rather more general motivational processes.

Behavioural activation and exertion of effort might be the most appropriate processes to account for the current results. During the appetitive phase of reward processing, animals are usually separated from the motivational stimuli by long distances, obstacles, or response costs. They put up with instrumental behaviour involving specific tasks to acquire the motivational stimuli (Salamone and Correa, 2012). The same assumption applies to humans performing the MID task. To maximise overall monetary outcome, gain and loss incentive cues require fast responding during target presentation which is accomplished with sustained attention and adaptive motor preparation during the whole experiment. Thus, sustained attention and motor preparation pose the adaptive response costs of MID tasks, i.e., they reflect effort spent on task. Consequently, behavioural activation describes a fundamental aspect of motivation since the regulation of motor programmes appears to operate under the control of neuronal systems directing behaviour towards or away from particular motivational objects (Salamone and Correa, 2012) — thereby accounting both for achieving a gain or avoiding a loss.

Extending beyond reward processing, dorsal and ventral striatum and its dopamine neurons have also been related to the processing of salient events by several research groups (Ravel et al., 1999, Setlow et al., 2003, Shimo and Hikosaka, 2001, Williams et al., 1993). More precisely, the NAcc might serve as part of the neuronal basis of the proposed behavioural activation mechanism. A recent connectivity study suggested that gain and loss anticipation involves an alerting signal of the thalamus converging interoceptive information provided by the insula to shape action selection programmes in the ventral striatum (Cho et al., 2012). In line with this finding, NAcc activation has also been found to correlate positively with stimulus salience (Zink et al., 2003), unpredictability of outcomes (Berns et al., 2001, Miller et al., 2014), and also with aversive stimuli (Delgado et al., 2011, Levita et al., 2009, Salamone, 1994, Salamone et al., 2007). These observations are in line with the present task since the valenced incentive cues had higher stimulus salience compared to the neutral cue and both yielded unpredictable outcomes. In addition, Mogenson et al. (1980) proposed that the NAcc can serve as a gate that translates motivation into motion. A recent study further supported this notion (Roesch et al., 2009) applying single-unit recordings in rats. This assumption would also fit to the present results. Both gain and loss incentive cues induced motivation to successfully perform the current task which led to subsequent target-induced motor responses. The P300 amplitude variation might reflect attention allocation towards task-relevant stimuli via evaluating rewarding and non-rewarding cues, whereas the ventral striatum activation during both incentive cues might reflect behavioural activation for the upcoming motor response. This would also explain the observed positive relation between P300 amplitudes and mean activation levels in the ventral striatum since both stem from the same underlying neuronal mechanism driving motivational processes.

## Limitations

The current MID task version was slightly modified compared to previous studies. The equiprobable outcomes after gain and loss incentive cues might have dampened the dopamine response after the incentive cues compared to previous studies (e.g., Knutson and Cooper, 2005, Knutson et al., 2001) which yielded a favourable outcome in approximately two thirds of all trials.

When conducting research combining fMRI and EEG, there is always the shortcoming that neuronal activity associated with cerebral blood flow and post-synaptic potentials have to be considered as distinct physiological processes assessing different aspects of the same underlying phenomenon. Several studies observed a linear relationship between fMRI and EEG measures suggesting that a common neuronal mechanism has to be reflected in both measurements (Logothetis, 2003, Sabatinelli et al., 2007), whereas others failed to do so (Nunez and Silberstein, 2000). Future studies should also address the current research question with simultaneous EEG–fMRI recordings to avoid potential state differences in arousal and mood of the participants between two timely separated sessions. Moreover, potential test–retest effects could be avoided by simultaneous EEG–fMRI measurements. However, in separating the two measurement sessions, the current study aimed to adapt and optimise the MID task explicitly to the specific requirements and constraints of each research method, resulting in more accurate and sensitive individual measurements.

The current study assumes that the observed effects were driven by dopamine-induced neuronal activation changes. However, fMRI and EEG only provide indirect evidence (if any) of dopaminergic transmission and effects. A direct assessment of neurotransmitter activity can be achieved by applying Positron Emission Tomography (PET) such as in a recent reward processing study (Urban et al., 2012). The combination of PET and fMRI or EEG might help to further disentangle the underlying neurochemical mechanisms of anticipation processes.

Future studies should also apply different established experimental paradigms for P300 investigation, such as the odd-ball task (Polich, 2007) to further investigate the relation between P300 amplitudes and activation levels in the ventral striatum, thereby varying the level of saliency of the administered stimuli and the effort necessary to perform the experimental task. Furthermore, the impact of different neurotransmitter systems on P300 amplitude variation should be addressed in future research.

## Conclusion

In summary, the present data showed that early stimulus evaluation processes within the first 600 ms after stimulus onset for all three incentive cues, reflected in P300 amplitude variation, are positively related to sustained activation levels in parts of the ventral striatum which is usually associated solely with “reward” processing. However, the current results point towards a more general motivational mechanism processing salient stimuli during anticipation. Reward processing might only be one aspect of this mechanism in action.
