# Supplementary material for: P300 amplitude variation is related to ventral striatum BOLD response during gain and loss anticipation: An EEG and fMRI experiment
Source: Neuroimage. 2014 Aug 1;96:12–21. doi: 10.1016/j.neuroimage.2014.03.077 (PMC4075343; doi:10.1016/j.neuroimage.2014.03.077)
Supplement: Supplementary file 1 — Supplementary material. [file mmc1.doc]

Supplemental Material

***P300 amplitude variation is related to ventral striatum BOLD response during gain and loss anticipation: An EEG and fMRI experiment***

1. **EEG-results/Feedback-Related Negativity (FRN)**

Previous EEG studies investigating the MID task did either not investigate cue-related ERPs prior to the P300 (Broyd et al., 2012) or interpreted the cue-related ERPs prior to the P300 to stimulus- and attention-related processes (Santesso et al., 2012). However, there is evidence that Feedback-related components such as the FRN can also be elicited by cues indicating upcoming outcomes. In general, the Feedback-Related Negativity (FRN; Miltner et al., 1997) is a negative-going component peaking around 200-300 ms after the presentation of external feedback. It is an ERP component that has been repeatedly used to investigate performance monitoring of external feedback. Larger FRN amplitudes have been reported after negative performance feedback (Miltner et al., 1997; Nieuwenhuis et al., 2004), after unexpected events (Hajcak et al., 2007; Pfabigan et al., 2011), after monetary losses (Gehring and Willoughby, 2002), and after salient compared to insignificant outcomes (Gehring and Willoughby, 2002; Yeung et al., 2005). Additionally, some studies found FRN-like ERPs when presenting predictive visual cues indicating upcoming feedback outcomes. Most of them used deterministic cues (Baker & Holroyd, 2009; Dunning & Hajcak, 2007; Krigolson & Holroyd, 2007), which are not comparable to the current MID task. However, a study by Liao et al. (2011) used probabilistic cues to assess ERPs prior to the actual feedback phase. Liao et al. (2011) observed that their predictive cues elicited smaller FRN components than unexpected outcomes, but marginally larger FRNs than expected outcomes. Incentive cue presentation of the current MID task was also probabilistic, negative feedback occurred approximately in 50% after each cue. Thus, one could speculate that these cues would also elicit FRN amplitudes, with most negative amplitude deflections after negative cues (Liao et al., 2011; Miltner et al., 1997).

Mean amplitudes were calculated for FRN amplitudes in the time window 250 – 300 ms after cue onset. The chosen time interval was based on visual inspection and literature recommendations (Broyd et al., 2012). FRN mean amplitudes were subjected to a repeated-measures ANOVA with the within-subject factors *electrode* (Fz, Cz, Pz) and *incentive cue* (gain, loss, neutral). This ANOVA yielded significant main effects for *electrode* (F(2,48)=96.07, p<0.001, η_p_^2^=0.80) and *incentive cue* (F(2,48)=29.93, p<0.001, η_p_^2^=0.56), but no significant interaction (F(4,96)=0.78, p=0.476). Amplitudes were most negative at Fz compared to Cz and Pz, and more negative at Cz than Pz (all p-values < 0.001). Moreover, amplitudes were most negative after neutral than loss and gain cues, and more negative after loss than gain cues (all p-values < 0.002).

The observed results support the interpretation that neutral cues were perceived as most unfavorable compared to gain and loss cues (Hajcak et al., 2005; Hajcak et al., 2006). This result again emphasizes that the neutral cue condition is no control condition. However, regarding FRN literature, one would rather expect negative cues, and not neutral cues, to elicit the largest amplitude deflections (Miltner et al., 1997). Thus, it is still somewhat speculative whether the negative deflection prior to the P300 component is an FRN component or rather an anterior N2 component indicating visual template matching or cognitive control (Folstein and Van Petten, 2008).

1. **ROI and correlational analysis with baseline contrasts**

We also extracted mean activation levels of the top 20% of all activated voxels from the following contrasts subtracting a baseline period (i.e., the fixation cross period prior to the anticipation cues) instead of the neutral anticipation cue (*gain cue > baseline*, *loss cue > baseline*, *neutral cue > baseline*) and calculated Pearson correlations with these values and the absolute ERP values. These correlations were calculated to further validate our correlational results.

Please note that we excluded one female participant from anatomical ROI correlational analysis because her BOLD mean activation levels were considered outliers by the PASW boxplot function.

*Anatomical ROI correlations:*

Gain anticipation P300 amplitudes correlated significantly with mean activation levels for the contrast *gain cue > baseline* in left ventral striatum (r=0.438, p=0.032) and by trend in right ventral striatum (r=0.365, p=0.080). Loss anticipation P300 amplitudes correlated significantly with mean activation levels for the contrast *loss cue > baseline* in left ventral striatum (r=0.450, p=0.027) and by trend in right ventral striatum (r=0.387, p=0.062). Neutral anticipation P300 amplitudes and mean activation levels for the contrast *neutral cue > baseline* were not correlated in left ventral striatum (r=0.267, p=0.207), but in right ventral striatum (r=0.472, p=0.020). No significant correlations emerged for anatomical ventral striatum ROI activation and CNV amplitudes for gain and loss cues (all p-values > 0.7676). For the neutral cue, the correlation between left ventral striatum activation levels and CNV amplitude did not reach significance (r=0.385, p=0.063), and was not significant for right ventral striatum (r=0.264, p=0.212).

*Functional ROI correlations:*

Gain anticipation P300 amplitudes correlated significantly with mean activation levels for the contrast *gain cue > baseline* in both left (r=0.506, p=0.010) and right (r=0.427, p=0.033) ventral striatum. Loss anticipation P300 amplitudes were by trend correlated with mean activation levels for the contrast loss cue > baseline in left ventral striatum (r=0.386, p=0.057). For right ventral striatum, a significant correlation was observed (r=0.449, p=0.024). Neutral anticipation P300 amplitudes and mean activation levels for the contrast neutral cue > baseline did not reach significance level in left (r=0.340, p=0.096) and right ventral striatum (r=0.155, p=0.459). No significant correlations emerged for functional ventral striatum ROI activation and CNV amplitudes for all three cues (all p-values > 0.319).

**Legends**

**Figure S1.** Left side: Difference wave amplitude courses for the comparisons gain cue > neutral cue and loss cue > neutral cue are depicted for CNV amplitudes at Fz (upper panel) and for P300 amplitudes at Pz (lower panel). Rectangles denote the respective time windows for analyses. Right side: Scalp topographies of the difference between gain cue > neutral cue and loss cue > neutral cue for the mean activation in the time window 650 – 1000 ms after stimulus onset for CNV component (upper panel), and in the time window 350 – 600 ms after stimulus onset for P300 component (lower panel).

**References**

Broyd, S.J., Richards, H.J., Helps, S.K., Chronaki, G., Bamford, S., Sonuga-Barke, E.J., 2012. An electrophysiological monetary incentive delay (e-MID) task: a way to decompose the different components of neural response to positive and negative monetary reinforcement. Journal of Neuroscience Methods 209, 40-49.

Folstein, J.R., Van Petten, C., 2008. Influence of cognitive control and mismatch on the N2 component of the ERP: A review. Psychophysiology 45, 152-170.

Gehring, W.J., Willoughby, A.R., 2002. The medial frontal cortex and the rapid processing of monetary gains and losses. Science 295, 2279-2282.

Hajcak, G., Holroyd, C.B., Moser, J.S., Simons, R.F., 2005. Brain potentials associated with expected and unexpected good and bad outcomes. Psychophysiology 42, 161-170.

Hajcak, G., Moser, J.S., Holroyd, C.B., Simons, R.F., 2006. The feedback-related negativity reflects the binary evaluation of good versus bad outcomes. Biological Psychology 71, 148-154.

Hajcak, G., Moser, J.S., Holroyd, C.B., Simons, R.F., 2007. It's worse than you thought: The feedback negativity and violations of reward prediction in gambling tasks. Psychophysiology 44, 905 912.

Liao, Y., Gramann, K., Feng, W., Deák, G.O., Li, H., 2011. This ought to be good: Brain activity accompanying positive and negative expectations and outcomes. Psychophysiology 48, 1412 1419.

Miltner, W.H.R., Braun, C.H., Coles, M.G.H., 1997. Event-related brain potentials following incorrect feedback in a time-estimation task: Evidence for a 'generic' neural system for error detection. Journal of Cognitive Neuroscience 9, 788-798.

Nieuwenhuis, S., Holroyd, C.B., Mol, N., Coles, M.G.H., 2004. Reinforcement-related brain potentials from medial frontal cortex: Origins and functional significance. Neuroscience and Biobehavioral Reviews 28, 441-448.

Pfabigan, D.M., Alexopoulos, J., Bauer, H., Sailer, U., 2011. Manipulation of feedback expectancy and valence induces negative and positive reward prediction error signals manifest in event related brain potentials. Psychophysiology 48, 656-664.

Santesso, D.L., Bogdan, R., Birk, J.L., Goetz, E.L., Holmes, A.J., Pizzagalli, D.A., 2012. Neural responses to negative feedback are related to negative emotionality in healthy adults. Social Cognitive and Affective Neuroscience 7, 794-803.

Yeung, N., Holroyd, C.B., Cohen, J.D., 2005. ERP correlates of feedback and reward processing in the presence and absence of response choice. Cerebral Cortex 15, 535-544.
